# GENDER DIFFERENCES IN COVID-19 EXPERIENCES OF OLDER FEMALE VETERANS USING THE PROSPECTIVE HERO CARE SURVEY DATA

**DOI:** 10.1093/geroni/igad104.3367

**Published:** 2023-12-21

**Authors:** Pranjal Tyagi, Sandra Garcia, Erin Bouldin, Ranak Trivedi, Orna Intrator, Mary Jo Pugh, Luci Leykum, Stuti Dang

**Affiliations:** South Florida VA Foundation for Research and Education (SFVAFRE), Miami, Florida, United States; University of Miami, Miami, Florida, United States; Decision-Enhancement and Analytic Sciences Center (IDEAS 2. .0.), Salt Lake City, Utah, United States; Stanford University, Stanford, California, United States; Canandaigua VAMC and University of Rochester, Canandaigua, New York, United States; VA Salt Lake City Health Care System, Salt Lake City, Utah, United States; South Texas Veterans Health Care System, San Antonio, Texas, United States; University of Miami, Miami, Florida, United States

## Abstract

Aging female Veterans faced unique experiences during the COVID-19 pandemic. We used self-reported experiences of older female Veterans during this time in comparison with male Veterans. We received responses for the HERO CARE survey from 2,080 (88 (4.2%) female, 1,992 (95.8%) male) community-dwelling Veterans from five geographically diverse Veterans Affairs Medical Centers. Veterans were prompted to self-report the impact of COVID-19 through an open-ended question asking to share anything they would like us to know about their experience. Open-ended responses were classified through inductive coding into emergent themes and chi-square tests. The most common emergent themes from female Veteran responses were social isolation (17.8%), negative effects on physical health (15.6%), and issues with accessing VA healthcare resources (15.6%). The most common emergent themes from male Veterans were social isolation (12.7%) and issues with accessing VA healthcare resources (8.2%). However, female Veterans were more likely to report experiencing a decline in their physical health compared to males (p< 0.01), more likely to report experiencing a negative change in their mental health (p< 0.01) and more likely to experience a decline in their general satisfaction with life (p< 0.002). They were also more likely to experience an issue with accessing VA healthcare resources (p< 0.02), and to experience financial problems (p< 0.0001). Female Veterans were more likely to report negative impacts of the COVID-19 pandemic to their mental and physical health, general satisfaction with life, and accessing VA healthcare resources. Routine assessment may be needed to adequately address their needs.

